# The Genetics of Hereditary Angioedema: A Review

**DOI:** 10.3390/jcm10092023

**Published:** 2021-05-09

**Authors:** Rosa Santacroce, Giovanna D’Andrea, Angela Bruna Maffione, Maurizio Margaglione, Maria d’Apolito

**Affiliations:** 1 Medical Genetics, Department of Clinical and Experimental Medicine, University of Foggia, 71122 Foggia, Italy; rosa.santacroce@unifg.it (R.S.); giovanna.dandrea@unifg.it (G.D.); maurizio.margaglione@unifg.it (M.M.); 2 Human Anatomy, Department of Clinical and Experimental Medicine, University of Foggia, 71122 Foggia, Italy; angelabruna.maffione@unifg.it

**Keywords:** HAE, C1-INH-HAE, nC1-INH-HAE, SERPING1, F12, PLG, ANGPT1, KNG1, MYOF, HS3ST6

## Abstract

Hereditary angioedema is a rare inherited disorder characterized by recurrent episodes of the accumulation of fluids outside of the blood vessels, causing rapid swelling of tissues in the hands, feet, limbs, face, intestinal tract, or airway. Mutations in SERPING1, the gene that encodes C1-INH (C1 esterase inhibitor), are responsible for the majority of cases of hereditary angioedema. C1 esterase inhibitor (C1-INH) is a major regulator of critical enzymes that are implicated in the cascades of bradykinin generation, which increases the vascular permeability and allows the flow of fluids into the extracellular space and results in angioedema. Moreover, a dominantly inherited disease has been described that has a similar clinical picture to C1-INH-HAE (Hereditary angioedema due to C1 inhibitor deficiency), but with normal C1-INH level and activity. This new type of HAE has no mutation in the SERPING1 gene and it is classified as nC1-INH-HAE (HAE with normal C1-INH). Currently mutations in six different genes have been identified as causing nC1-INH-HAE: factor XII (F12), plasminogen (PLG), angiopoietin 1 (ANGPT1), Kininogen 1 (KNG1), Myoferlin (MYOF), and heparan sulfate (HS)-glucosamine 3-O-sulfotransferase 6 (HS3ST6). In this review we aim to summarize the recent advances in genetic characterization of angioedema and possible future prospects in the identification of new genetic defects in HAE. We also provide an overview of diagnostic applications of genetic biomarkers using NGS technologies (*Next Generation Sequencing*).

## 1. Introduction

Angioedema is characterized by a localized, self-limiting, and transient subcutaneous or submucosal swelling, which can present with or without episodes of urticaria, and usually subsides within 24–37 h. The clinical expression is highly variable, from asymptomatic individuals to patients suffering from disabling and life-threatening attacks with a demonstrated humanistic and economic burden [1]. Manifestations may imply swelling of the extremities and superficial regions of the face; affect the gastrointestinal tract, because of edema of the bowel wall, and cause severe cramping abdominal pain, nausea, vomiting and, sometimes, diarrhea; and occasionally compromise breathing, including laryngeal edema and/or severe tongue/pharyngeal edema so that secretions cannot be handled [2]. The severity and frequency of acute attacks of angioedema are variable, ranging from once/year to three attacks/week [3]. Although a series of predisposing circumstances, including trauma, fluctuating hormone level (particularly increased estrogen), infection, and severe emotional stress have been identified, most acute episodes of angioedema seem apparently spontaneous. In the general population, an acute episode of angioedema has been estimated to occur in up to 7.4% of subjects during their lifetime [4]. Thus, after excluding cases of angioedema with identifiable cause or wheals, one person in a hundred is likely to have an episode of angioedema. Approximately 0.05% of them have been estimated to suffer from recurrent/nonallergic angioedema [5].

The diagnosis is based on the clinical description, the identification of triggers for the attacks, the response to medications during an acute episode, and possible familial history. Depending on the results of history, physical examination and laboratory investigations, it may be classified as drug-induced angioedema, hereditary angioedema, or acquired angioedema.

## 2. Hereditary Angioedema

Hereditary angioedema (HAE) is a genetic disorder that predisposes an individual to develop vasogenic edema. Prevalence of HAE has been reported to be 1 in 10,000 to 1 in 150,000 [6]. HAE shows no ethnic- or sex-based differences but tends to be more severe in women [2,7]. The pathogenesis of HAE involves the accumulation of extravascular fluid in various tissues via a non-inflammatory and non-allergic mechanism. Clinical manifestations include abrupt onset swelling around the eyes, face and extremities; pain in theabdomen (as a result of bowel edema), and laryngeal edema leading to hoarseness of voice, breathing difficulty, and occasionally death [8,9].

Several disorders may manifest with subcutaneous or submucosal swelling. The presence of severe swelling can be mistaken for an allergic reaction or acute abdominal condition. Misdiagnosis can lead to ineffective therapies and unnecessary surgeries [10]. In 1963, Donaldson and Evans discovered that HAE was caused by a genetic deficiency of the C1 inhibitor (C1-INH) [11]. Since then, many large studies have established that the most common type of hereditary angioedema (HAE) is the result of gene mutations resulting in reduced C1-INH functional plasma. This results in two HAE variants of C1-INH-HAE: type 1 and type 2. C1-INH type 1 results from a failure to synthesize C1-INH, whereas in type 2, an abnormal, non-functional protein is synthesized.

**Figure 1:**
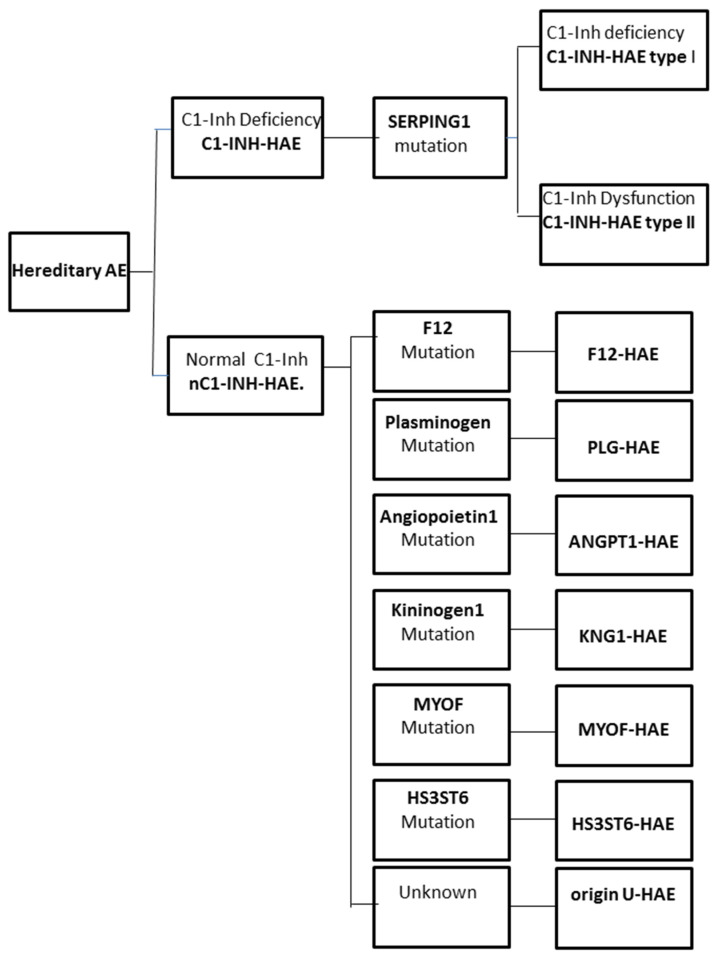
Classification of the different forms of hereditary angioedema (HAE).

The increase in vascular permeability that causes angioedema in HAE is related to the mediators of the contact system or kallikrein–kinin pathway. C1-INH regulates the contact system through the inhibition of plasma kallikrein and coagulation factor FXIIa. The loss of the inhibitory activity of C1-INH leads to bradykinin overproduction. It has been documented that an increased release of bradykinin is the cause of angioedema via its action on B2 receptors leading to an increase in vascular permeability [12,13]. Moreover, a dominantly inherited disease has been described that has a similar clinical picture to C1-INH-HAE, but with normal C1-INH level and activity. This new type of HAE has no mutation in the SERPING1 gene and it is classified as nC1-INH-HAE (Figure 1).

### 2.1. Genetics of C1-INH-HAE

C1-INH-HAE is the most frequent type of HAE due to low production or nonfunctional serine protease inhibitor, namely C1 esterase inhibitor (C1-INH). The autosomal-dominant mode of inheritance has been described for C1-INH-HAE when one of the two alleles of the C1-INH gene (SERPING1) is mutated [14,15,16].

**Figure 2:**
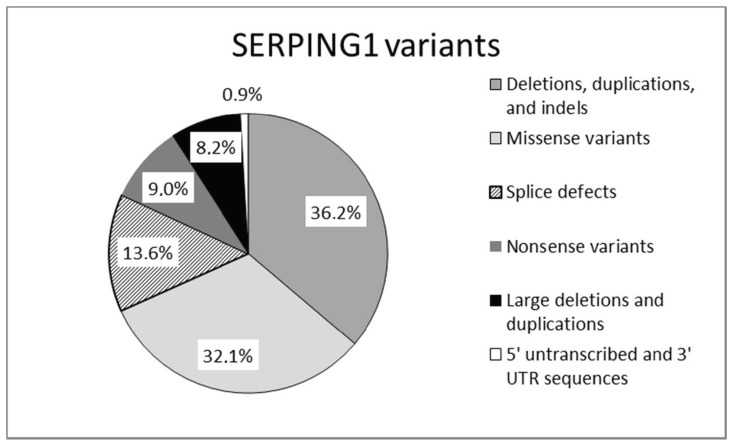
Mutations reported in SERPING1 gene [17].

Mutations in SERPING1, the gene that encodes for C1-INH, are responsible for the majority of cases of hereditary angioedema. Mutations in the SERPING1 gene may be classified as shown in Figure 2.

Mutations leading to type 1 are dispersed throughout the entire SERPING1. Type 2 mutations map around the protein reactive center loop (RCL), with the single exception of a mutation in the amino acid residue Lys251, which affects functionality after protein folding [15,16]. This gene is characterized by a great allelic heterogeneity and approximately 748 different mutations have been reported and collected in the Human Gene Mutation Database (HGMD) and in a specific, online HAE database (HAEdb, hae.enzim.hu) [17,18,19]. The vast majority were identified as heterozygous variants (729), though homozygous variants in 10 probands and compound heterozygous variants (nine combinations) were found. In addition, probands were identified as de novo cases and six probands with heterozygous variants exhibited gonadal mosaicism [17].

The severity and course of HAE may vary greatly even among family members harboring the same mutation. A correlation between different types of mutations and clinical phenotype is controversial. Only patients carrying missense mutations leading to the change of a single amino acid exhibited a less severe clinical phenotype [20]. The involvement of epigenetic changes [21] and environmental factors (i.e., temperature, pH, and oxidative stress) in the pathogenesis of HAE, has also been postulated [16].

Recently the advent of new technology allowed the identification of novel SERPING1 variants in C1-INH-HAE patients in particular pathogenic intronic regions. These are hardly detectable with a direct sequencing approach. Using NGS technology two pathogenic deep intronic variants have been detected: the c.-22- 155G > T located at intron 1 and the c.1029 + 384A > G located at intron 6 [22,23,24].

### 2.2. The Genetics of nC1-INH-HAE

In 2000, separately Bork et al. [25] and Binkley et al. [26] described another type of HAE with a quantitatively and qualitatively normal C1-INH (labeled as nC1-INH-HAE). In this rare type of HAE, clinical manifestations were similar to C1INH deficiency HAE, except that there is a higher female predominance due to aggravation during pregnancy and estrogen dependency. In these groups of patients no mutation was detected in the SERPING1 gene. In 2006 two missense mutations (p.Thr328Lys and p.Thr328Arg) in the F12 gene were detected for the first time in six out of twenty German families with nC1-INH-HAE [27]. Other rare pathogenic mutations in this gene have been identified at the same locus in different cohorts: F12 gene encoding for the coagulation factor XII. HAE-FXII is inherited as an autosomal dominant trait with incomplete penetrance and is the result of a gain-of-function mutation in the F12 gene. All of these mutations occur at exon 9 of F12 which encodes a highly glycosylated region of the protein and leads to an increased production of activated Factor XII (Factor XIIa) via activation by plasmin [27,28,29,30,31,32].

Table 1 summarizes the mutations described in F12 and in the other genes associated with nC1-INH-HAE.

In the following years families or individuals with nC1-INH-HAE, in particular families with nC1-INH-HAE and without mutation in the F12 gene, were analyzed often by whole-exome sequencing (WES), but also by a direct candidate gene approach [33,34,35,36,37,38,39]. These studies allowed the identification of five new genes linked nC1-INH-HAE. In 2018 a novel variant in the plasminogen gene (PLG) was described in patients with nC1-INH-HAE [33,34]. The mutation c.988A > G, located in exon 9 of the PLG gene, leads to the missense mutation p.Lys330Glu (K330E) in the kringle 3 domain of the PLG protein. In large multiple-case families, co-segregation of the mutation with the disease phenotype was demonstrated; the inheritance pattern was autosomal dominant, with incomplete penetrance [33,34]. This mutation has been reported in more than 14 patients with nC1-INH-HAE belonging to 4 families and was transmitted as an autosomal dominant trait presenting incomplete penetrance [33]. This mutation has been reported in more than 125 affected individuals from different countries [40]. Plasminogen is the inactive precursor protein of the enzyme plasmin. Plasmin plays a role in bradykinin production via activation of factor XII. The mutant protein leads to the increased production of the bradykinin.

In the same year, in affected female members of a large nC1-INH-HAE family, a missense variant in the angiopoietin-1 gene (ANGPT1), c.355G > T (p.Ala119Ser), was identified [35]. This variation disrupts the multimerization of the protein and affects the protein’s abilities to bind its specific receptor on endothelial cells suggesting a novel and independent mechanism leading to vascular permeability and angioedema through a mechanism of haploinsufficiency [41]. Other potentially pathogenic variants of ANGPT1 (p.Ala8Val; p.Gln370His) were found by Cagini et al. [42].

Recently, WES has led to the identification of another two causal variants in two nC1-INH-HAE families. Bork et al. [36] identified a novel variant of the KNG1 gene encoding the high molecular weight as well as low molecular weight kininogen (proteins). The novel mutation (p.Met379Lys) resulted in the substitution of a methionine with lysine at position 379 and co-segregated with clinical symptoms of hereditary angioedema (HAE) with normal C1-INH levels in three generations of a large German family.

In 2020 in an Italian family, a rare Myoferlin variant (MYOF Arg217Ser) was identified and three out of four carriers were symptomatic. The MYOF-217S variant acts with a gain of function mechanism and allows a higher ability of the mutant protein to localize VEGFR-2 to the plasma membrane in response to VEGF stimuli, which suggests the involvement of VEGF-mediated signaling in HAE. The identification of the MYOF-217S variant as causative of HAE suggests an involvement of the VEGF-mediated signaling in HAE pathophysiology [38].

By performing whole exome sequencing on a multigenerational family with nC1-INH-HAE, a mutation in the heparan sulfate (HS)-glucosamine 3-O-sulfotransferase 6 (HS3ST6) has been identified. This mutation, c.430A > T (p.Thr144Ser), has been found in all three affected family members. This gene encodes HS-glucosamine 3-O-sulfotransferase 6 (3-OST-6), which is involved in the last step of HS biosynthesis. The p.Thr144Ser mutation is hypothesized to result in incomplete HS biosynthesis and is likely to affect cell surface interactions of key players in angioedema formation and represent a novel mechanism for disease development [39].

### 2.3. Disease-Modifying Factors

The high variability in clinical expression between patients with the same mutation led to the hypothesis that mutations in these and other related genes could be potential modifiers in the clinical phenotype of patients with known genetic causes of HAE. Several polymorphisms in different genes and their effects on the clinical phenotype of patients were analyzed. These included p.Y244C, p.G354R, and p.T916M in the ACE gene; p.C548Y in the KLKB1 (kallikrein) gene; and p.D287N in the NOS3 (nitric oxide synthase) gene [43]. However, the role of these polymorphisms on modification in the clinical phenotype of HAE remains to be clarified. Other disease-modifying factors, F12 or KLKB1 gene polymorphisms, have been studied to explain clinical variability of C1-INH-HAE or nC1-INH-HAE. The possible associations of the functional promoter F12-46C/T polymorphism (rs1801020) with clinical features of C1-INH-HAE and the SERPING1 mutational status have been investigated. The F12-46C/T carriage acts as an independent modifier of C1-INH-HAE severity regardless of SERPING1 mutational status [44,45]. The c.-4C/T polymorphism (rs1801020) in the 5-UTR region of the F12 gene was shown to significantly influence the degree of contact system activation and the clinical severity of the disease. Patients carrying FXII-HAE (p.Thr309Lys variant) and c.-46CC genotype exhibited more severe and frequent manifestations of the disease [46].

The c.428G/A (rs3733402) polymorphism in the KLKB1 gene, encoding plasma kallikrein, was also investigated. Carriers of the G allele of the KLKB1-428G/A polymorphism exhibited a significantly delayed disease onset [47]. Since an earlier onset of symptoms is inversely correlated with the subsequent course of the disease, these polymorphisms may be helpful prognostic biomarkers of disease severity.

## 3. Diagnostic Applications of Genetic Biomarkers

The implementation of NGS technologies, genome-wide sequencing, and especially whole exome sequencing (WES) allows the identification of additional genes not previously recognized as nC1-INH HAE. In addition to its role in research, NGS also represents a very important tool in clinical diagnostics. In approximately 5% of C1-INH-HAE patients, no causal mutation is identified with the standard mutational screening of the coding exons and exon-intron boundaries in the SERPING1 gene. Conventional methods for genotyping of C1-INH-HAE patients do not allow the analysis of SERPING1 intronic regions. Recently, with a custom next-generation sequencing (NGS) platform, it has been possible to analyze SERPING1 in its full length and identify two pathogenic deep intronic variants: c.-22- 155G > T located at intron 1 and c.1029 + 384A > G located at intron 6 [22,23]. However, SERPING1 genotyping is not recommended for the diagnosis of C1-INH-HAE because C1-INH-HAE presents a great allelic heterogeneity, and biochemical C1-INH testing is cost-effective and reliable.

The understanding of the genetic basis of nC1-INH HAE allows for improvement of diagnosis by using a mutation analysis approach. Genotyping is required for the diagnosis of nC1-INHHAE [43]. Since four F12 alterations in exon 9 have been identified, only exon 9 of F12 should be investigated as a routine molecular diagnostic biomarker of FXII-HAE. The pathogenic PLG, ANGPT1, KNG1, MYOF, or HS3ST6 variants, in addition to variants in F12, could serve as diagnostic biomarkers for patients with unexplained angioedema, providing a molecular-level assay for establishing a diagnosis of HAE with normal C1-INH. In addition, it is conceivable that beyond the genes already associated with HAE, there are many other candidate disease genes remaining to be examined, including many endothelium-associated ones. [48]

Identification of new genes and families with HAE with normal C1-INH will facilitate the diagnosis of additional patients and the study of the mechanisms of pathogenesis of this set of disorders. Understanding the disease pathophysiology could allow for the identification of new therapeutic targets and improve clinical tools for the management of the disease.

## Figures and Tables

**Table 1:** Summary of the mutations in nC1-INH-HAE.

F12	T328K T328R c.971_1018 + 24del72 c.892_909dup
PLG	K330E
ANGPT1	A119S A8V Q370H
KNG1	M379K
MYOF	R217S
HS3ST6	T144S

## Data Availability

This statement can be excluded.
